# Supplementary material for: Hybrid Models and Biological Model Reduction with PyDSTool
Source: PLoS Comput Biol. 2012 Aug 9;8(8):e1002628. doi: 10.1371/journal.pcbi.1002628 (PMC3415397; doi:10.1371/journal.pcbi.1002628)
Supplement: Text S4 — Complete source code for the PyDSTool package (version 0.88.120504). Includes API documentation and help files linking to web pages. This file is identical to the current public release on Sourceforge.net. (ZIP) [file pcbi.1002628.s004.zip › PyDSTool/html/PyDSTool.PyCont.Continuation-module.html]

xml version="1.0" encoding="ascii"?


PyDSTool.PyCont.Continuation


| Home | Trees | Indices | Help | PyDSTool |
| --- | --- | --- | --- | --- |

Package PyDSTool ::
Package PyCont ::
Module Continuation

[hide private]

[frames] | no frames]

# Module Continuation

source code

Curve classes: Continuation, EquilibriumCurve, FoldCurve,
HopfCurveOne, HopfCurveTwo

Drew LaMar, March 2006

Continuation is the ancestral class of all curve classes and contains
the continuation algorithms (Moore-Penrose, etc.) It also contains all
methods that are general to any curve found using continuation.


|  |  |
| --- | --- |
| Classes | [hide private] |

Continuation  
Abstract continuation class

EquilibriumCurve  
Child of Continuation class that represents curves of
equilibrium points.

FoldCurve  
Child of Continuation class that represents curves of limit
points.

HopfCurveOne  
Child of Continuation class that represents curves of Hopf
points.

HopfCurveTwo  
Child of Continuation class that represents curves of Hopf points.

FixedPointCurve

LimitCycleCurve  
Wrapper for auto limit cycle computations.

UserDefinedCurve  
User defined curve.


|  |  |
| --- | --- |
| Functions | [hide private] |

|  |  |
| --- | --- |
| random(size=None)  Return random floats in the half-open interval [0.0, 1.0). | source code |


|  |  |
| --- | --- |
| Variables | [hide private] |

FLOQ\_TOL

conjugate

negate

clock

sum

sqrt

evec1\_standard

argmax

ndarray

PI

multiply

identity

pointsToPointset

log10 = `<ufunc 'log10'>`

Inf = `inf`

NaN = `nan`

isfinite = `<ufunc 'isfinite'>`

r\_ = `<numpy.lib.index_tricks.RClass object at 0x11542f0>`

c\_ = `<numpy.lib.index_tricks.CClass object at 0x1154330>`

sign = `<ufunc 'sign'>`

mod = `<ufunc 'remainder'>`

subtract = `<ufunc 'subtract'>`

divide = `<ufunc 'divide'>`

isnan = `<ufunc 'isnan'>`

\_classes = `['Continuation', 'EquilibriumCurve', 'FoldCurve', '...`

\_constants = `['cont_args_list', 'cont_bif_points', 'equilibriu...`

cont\_args\_list = `['name', 'force', 'freepars', 'MaxNumPoints',...`

cont\_bif\_points = `['BP', 'B', 'SP']`

equilibrium\_args\_list = `['LocBifPoints']`

equilibrium\_bif\_points = `['LP', 'H']`

fold\_args\_list = `['LocBifPoints']`

fold\_bif\_points = `['BT', 'ZH', 'CP']`

hopf\_args\_list = `['LocBifPoints']`

hopf\_bif\_points = `['BT', 'ZH', 'GH', 'DH']`

fixedpoint\_args\_list = `['LocBifPoints', 'period']`

fixedpoint\_bif\_points = `['PD', 'LPC', 'NS']`

limitcycle\_args\_list = `['LocBifPoints', 'NumCollocation', 'Num...`

limitcycle\_bif\_points = `['PD', 'LPC', 'NS']`

userdefined\_args\_list = `['LocBifPoints']`

other\_special\_points = `['RG', 'UZ', 'P', 'MX']`

auto\_point\_types = `{-9: 'MX', -4: 'UZ', 1: 'BP', 2: 'LP', 3: '...`

solution\_measures\_list = `['max', 'min', 'avg', 'nm2']`

solution\_measures = `{'avg': 1, 'max': 0, 'min': 0, 'nm2': 2}`

all\_args\_list = `['force', 'NumIntervals', 'SPOut', 'period', '...`

all\_point\_types = `['NS', 'B', 'ZH', 'PD', 'DH', 'UZ', 'H', 'SP...`

all\_curve\_types = `['EP-C', 'LP-C', 'H-C1', 'H-C2', 'FP-C', 'LC...`

bif\_curve\_colors = `{'EP-C': 'k', 'FP-C': 'g', 'H-C1': 'b', 'H-...`

bif\_point\_colors = `{'B': 'dr', 'BP': 'og', 'BT': 'sy', 'CP': '...`

stab\_line\_styles = `{'N': '-.', 'S': '-', 'U': '--', 'X': ':'}`


|  |  |
| --- | --- |
| Function Details | [hide private] |

|  |  |
| --- | --- |
| random(size=None) | source code |

```
Return random floats in the half-open interval [0.0, 1.0).

Results are from the "continuous uniform" distribution over the
stated interval.  To sample :math:`Unif[a, b), b > a` multiply
the output of `random_sample` by `(b-a)` and add `a`::

  (b - a) * random_sample() + a

Parameters
----------
size : int or tuple of ints, optional
    Defines the shape of the returned array of random floats. If None
    (the default), returns a single float.

Returns
-------
out : float or ndarray of floats
    Array of random floats of shape `size` (unless ``size=None``, in which
    case a single float is returned).

Examples
--------
>>> np.random.random_sample()
0.47108547995356098
>>> type(np.random.random_sample())
<type 'float'>
>>> np.random.random_sample((5,))
array([ 0.30220482,  0.86820401,  0.1654503 ,  0.11659149,  0.54323428])

Three-by-two array of random numbers from [-5, 0):

>>> 5 * np.random.random_sample((3, 2)) - 5
array([[-3.99149989, -0.52338984],
       [-2.99091858, -0.79479508],
       [-1.23204345, -1.75224494]])
```

  


|  |  |
| --- | --- |
| Variables Details | [hide private] |

### \_classes

Value:
:   ```
    ['Continuation',
     'EquilibriumCurve',
     'FoldCurve',
     'HopfCurveOne',
     'HopfCurveTwo',
     'FixedPointCurve',
     'LimitCycleCurve',
     'UserDefinedCurve']
    ```

### \_constants

Value:
:   ```
    ['cont_args_list',
     'cont_bif_points',
     'equilibrium_args_list',
     'equilibrium_bif_points',
     'fold_args_list',
     'fold_bif_points',
     'hopf_args_list',
     'hopf_bif_points',
    ...
    ```

### cont\_args\_list

Value:
:   ```
    ['name',
     'force',
     'freepars',
     'MaxNumPoints',
     'MaxCorrIters',
     'MaxTestIters',
     'MaxStepSize',
     'MinStepSize',
    ...
    ```

### limitcycle\_args\_list

Value:
:   ```
    ['LocBifPoints',
     'NumCollocation',
     'NumIntervals',
     'AdaptMesh',
     'NumSPOut',
     'DiagVerbosity',
     'SolutionMeasures',
     'SaveFlow']
    ```

### auto\_point\_types

Value:
:   ```
    {-9: 'MX',
     -4: 'UZ',
     1: 'BP',
     2: 'LP',
     3: 'H',
     4: 'RG',
     5: 'LPC',
     6: 'BP',
    ...
    ```

### all\_args\_list

Value:
:   ```
    ['force',
     'NumIntervals',
     'SPOut',
     'period',
     'ClosedCurve',
     'SolutionMeasures',
     'NumSPOut',
     'SaveEigen',
    ...
    ```

### all\_point\_types

Value:
:   ```
    ['NS',
     'B',
     'ZH',
     'PD',
     'DH',
     'UZ',
     'H',
     'SP',
    ...
    ```

### all\_curve\_types

Value:
:   ```
    ['EP-C', 'LP-C', 'H-C1', 'H-C2', 'FP-C', 'LC-C']
    ```

### bif\_curve\_colors

Value:
:   ```
    {'EP-C': 'k',
     'FP-C': 'g',
     'H-C1': 'b',
     'H-C2': 'b',
     'LC-C': 'm',
     'LP-C': 'r',
     'UD-C': 'k'}
    ```

### bif\_point\_colors

Value:
:   ```
    {'B': 'dr',
     'BP': 'og',
     'BT': 'sy',
     'CP': 'sr',
     'DH': 'sg',
     'GH': 'sb',
     'H': 'ob',
     'LP': 'or',
    ...
    ```

  


| Home | Trees | Indices | Help | PyDSTool |
| --- | --- | --- | --- | --- |

|  |  |
| --- | --- |
| Generated by Epydoc 3.0.1 on Fri May 4 15:24:03 2012 | http://epydoc.sourceforge.net |
